# Supplementary material for: Pea Protein-Derived Peptides Inhibit Hepatic Glucose Production via the Gluconeogenic Signaling in the AML-12 Cells
Source: Int J Environ Res Public Health. 2022 Aug 18;19(16):10254. doi: 10.3390/ijerph191610254 (PMC9408102; doi:10.3390/ijerph191610254)
Supplement: Supplementary file 1 [file ijerph-19-10254-s001.zip › ijerph-1855296-supplementary.pdf]

Supplementary Table S1: Peptide profile of PPH

Peptide sequence	Molecular weight (kDa)
RPVKEL	741.46175
NLPLDVV	769.44543
SKITPKD	788.45124
KVEDGLH	797.41519
EKNVIVK	829.51418
VKPPIIY	829.5182
EENVIVK	830.46181
NEKSKLL	831.49344
PNHPELK	834.44683
TPLFPRI	843.5087
SHKPEYS	847.39446
VERPVKE	856.48869
KEEVKVE	860.47237
TDEEFAR	867.38429
EPDHRVE	881.41117
NNERNFL	906.44281
YEEIEKV	909.45639
REQIEEL	916.47344
KRDFLED	922.46287
DYVYLPR	925.47779
REDPEER	930.42755
NEPWWPK	956.46248
RERWERE	1060.52827
DTIKLPAG	814.46689
PVNGPGKF	815.44101
PENGLGKL	827.46214
RATPGEVL	842.47304
PVNKPGQL	852.49378
LSPLPVVK	852.55532
PDAPLDKV	854.46181
EGSLLLPH	865.47779
VRPPPLTS	866.50943
DADLIVPK	870.49311
LSEKIAPL	870.52949
KLTPGDVF	876.48254
PVNRPGQL	880.49993
NLERGDAI	887.45812
KGELETVL	888.50367
IKAPADKF	889.51418
INPVLLPK	893.58186

SDLPKLVE	900.50367
IISPPEKQ	911.51966
PVNRPGKF	914.52066
GQTPLFPR	915.50468
TLDDIPNH	924.44214
NPNNPELK	925.47377
NQLDSTPR	930.46393
SNKDKIIL	930.56186
LDSTPRVF	934.49926
IENPVKEL	941.53022
KYPPPKVI	941.58186
KEVQPGRE	942.50032
NPNHPELK	948.48976
SPNDRNSY	952.4119
EVEEVIKN	959.5044
RSDPQNPF	960.45337
ENENGHIR	968.45443
VERPVKEL	969.57276
DNEDLRVL	973.4949
REGTDFVR	979.49557
IQRPVKEL	982.60439
RIDLPEKL	983.58841
NRDDNEDL	990.41229
TPLFPRIF	990.57711
RSDQENPF	992.4432
TDEEFARE	996.42688
NQLDQMPR	1001.48329
SREQIEEL	1003.50546
DDEEDLRL	1004.45309
KDEFPDVR	1005.49999
RDFLEDAF	1012.47344
SDYVYLPR	1012.50982
REEELNNL	1016.50071
EFEPINLR	1017.53637
LRSRNPIY	1018.57924
YEEIEKVL	1022.54045
TRSEFDRL	1023.52178
PEERARLR	1026.5803
RDDNEDLR	1032.47048
ENNERNFL	1035.4854
REQIEELK	1044.5684
RDENEKLY	1066.51636
REQIEELR	1072.57455

ERRNPFLF	1078.57924
KDKPWWPK	1084.59383
RREDPEER	1086.52866
KEEFFFPY	1106.51932
IGTPGKGIL	855.52983
VAKPKPGSS	870.50434
GDTIKIPAG	871.48836
RGDAIKLPA	940.55744
VNDGGIIEK	944.50474
GPTPVRDGF	945.47886
GIKAPADKF	946.53564
TLSPLPVVK	953.60299
NLPAIPHPT	959.53089
GEETDAIVK	961.48367
TGEGDEKVQ	962.44253
APSKDAPMF	963.46043
IPVNKPGQL	965.57784
RGDTIKIPA	970.56801
EDAITIPGR	971.51564
YEGGIKLPT	977.53022
PVNKPGQLQ	980.55236
EGKGDFELV	993.48875
KISPLPVLK	994.66593
SIISPPEKQ	998.55169
PVNRPGQLQ	1008.5585
IKEKIPGTE	1014.58299
PVRDGFKSL	1018.56801
LGGEDFDNR	1022.45376
KVEDGLHII	1023.58332
ERGDTIKLP	1028.57348
SEDPDVDVR	1031.46399
VERPVKELA	1040.60987
GHYPNPDIE	1041.4636
PQDERGQIV	1041.53235
KGQTPLFPR	1043.59964
SQEKNVIVK	1044.60479
SQEENVIVK	1045.55242
SRSDPQNPF	1047.4854
DDQIMDGVR	1048.47278
ALEPDNRIE	1056.53201
KIDEEGRIV	1058.58405
VVAPPERKY	1058.59931
NTEEDTAKR	1063.50144

DDQIMDGVR	1064.4677
ALEPDHRVE	1065.53235
KYPPPKVIQ	1069.64044
RSPQDERGQ	1072.51301
RNDEELGKL	1073.55856
APKKSLWPF	1073.61423
NLEEGDIMR	1076.50408
ENENGHIRL	1081.5385
DDNEDLRVL	1088.52184
DDEEDLRVV	1089.50586
SDQENPFIF	1096.49457
QVQRPVKEL	1096.64732
DSTPRVFYL	1097.56259
KEVQPGRER	1098.60143
LPRQPSELY	1102.58914
VNRDDNEEL	1103.49636
SEPFNLRSR	1105.57488
VYLPRDEAF	1109.56259
RQLPRQPSE	1110.60143
WNPNNPELK	1111.55308
RNPEEIPWA	1111.55308
SRDPLKLPW	1111.62585
DWKETPEAH	1112.50071
DHHDSIMPY	1114.46222
FENENGHIR	1115.52285
NNQLDQMPR	1115.52622
RKEKDPELT	1115.60551
SVREDQRIL	1115.61675
REGEEEERS	1120.48652
VAEDRTERF	1122.55381
RLPPLEEIR	1122.66297
TDYEEIEKV	1125.53101
LDIDRLIRL	1126.69427
TDEEFAREM	1127.46736
YENENGHIR	1131.51776
RDDNEDLRV	1131.53889
NLRSRNPIY	1132.62217
NQDDEEDLR	1133.47054
WNPNHPELK	1134.56907
YEEIEKVLL	1135.62452
NYEEIEKVL	1136.58338
DYEEIEKVL	1137.5674
RNPIYSNKF	1138.60037

DLPVLRWLK	1139.69354
NTDYEEIEK	1140.50552
KRDFLEDAF	1140.5684
LEEHEKETQ	1142.53241
TDEEFAREM	1143.46228
NRDDNEDLR	1146.5134
REEDEEQVD	1148.4702
LEEHEKETH	1151.53274
ENLQNYRLL	1162.6215
RSDYVYLPR	1168.61093
EIRVPWEIK	1169.66772
REEELNNLR	1172.60182
YREEELNNL	1179.56404
EEEEEEIQR	1190.51715
EDEEEKQKY	1197.52699
KDKPWWPKL	1197.67789
DKNDEWHRV	1198.55996
RERWEREED	1304.5978
IGTPGKGILA	926.56694
YIGTPGKGIL	1018.59316
RGDTIKLPAG	1027.58947
VIPVNGPGKF	1027.59349
SVEKKDPTGA	1031.53677
AIPVNKPGQL	1036.61496
NIKGRGIIGL	1040.65749
KAKLSSGDVF	1051.57824
DEGSEPRVPA	1056.49563
GDSIGEEVEK	1062.49496
AIPVNRPGQL	1064.6211
TIPVNKPGQL	1066.62552
EGSEPRVPAQ	1069.52726
SIPTKPIEGQ	1069.5888
ERGDAIKLPA	1069.60003
REGDIIAVPT	1070.58405
GPTPVRDGFK	1073.57382
IGAPDMAFPR	1074.54008
INPDAPLDKV	1081.5888
SQISPLPVLK	1081.66157
HNPGGPNEDF	1083.44901
LGNPDHGEHL	1088.51195
YEGGIKLPTN	1091.57315
IPVNKPGQLQ	1093.63642
GAPEDEIRHA	1094.52251

TVNEGKGDFE	1095.49529
ERGDTIKIPA	1099.6106
NEDEEKGAIV	1103.52151
GRNEDEEKGA	1104.49161
SLPTEPLHPN	1104.5684
NLPAIPHPTF	1106.59931
LSIISPPEKQ	1111.63575
LERGDAIKLP	1111.64698
IANQLDSTPR	1114.58511
FEEGLPPILT	1115.5983
FIAPVDTKPQ	1115.60954
TPVRDGFKSL	1119.61568
IPVNRPGQLQ	1121.64257
NKRIPVINPA	1121.67895
SNNPAFSNKF	1125.53235
NKDKIILGPK	1125.69902
VIPVNRPGKF	1126.67314
SPQDERGQIV	1128.56438
DKIKEKLPGT	1128.6623
SLTLPILRNL	1139.71467
RRLLPEEGTA	1141.6324
LERGDTIKLP	1141.65755
TVTVPKEEIK	1143.66197
TLNQLDSTPR	1144.59568
TLPGDEVERM	1146.54595
TLPVLKLLHL	1146.76089
TDDQIMDGVR	1149.52046
TFNTEEDTAK	1155.51642
SFPRENPAFV	1163.58438
NALEPDNRIE	1170.57494
DALEPDNRIE	1171.55896
VRNDEELGKL	1172.62698
ELPPTHPIRL	1172.67862
SLTLPVLRYL	1174.71942
GQTPLFPRIF	1175.65716
DDQIMDGVRK	1176.56775
NTEEDTAKRL	1176.58551
NQLDSTPRVF	1176.60076
NALEPDHRVE	1179.57528
SQVERPVKEL	1184.66336
RSPQDERGQI	1185.59707
EINEENVIVK	1186.63139
VLKPDDRNSF	1190.61641

DDQIMDGVRK	1192.56266
SQIQRPVKEL	1197.695
SSRDPLKLPW	1198.65788
KVSKKQIEEL	1201.71506
SNNQLDQMPR	1202.55825
DLAKNKNQYL	1206.64771
REGEEEERSS	1207.51855
VDWKETPEAH	1211.56913
TWNPNNPELK	1212.60076
GEEEEELEKE	1220.51648
RSDPQNPFIF	1220.60585
AEKVDEVFER	1221.61099
RQLPRQPSEL	1223.68549
SDQENPFIFK	1224.58953
IDWKETPEAH	1225.58478
SETYPYPRRG	1225.59601
GSETYPYPRR	1225.59601
SRNPIYSNKF	1225.6324
DHHDSIMPYL	1227.54628
FENENGHIRL	1228.60691
LFENENGHIR	1228.60691
LVNRDENEKL	1229.64844
KVSREQIEEL	1230.66884
VNQDDEEDLR	1232.53895
EITPEKKYPQ	1232.65213
YVPRDENFGH	1233.56471
LDEQKKGRIF	1233.695
GKEEEEEKEQ	1234.54337
TWNPNHPELK	1235.61675
DKRSDLFENL	1236.62189
TNYEEIEKVL	1237.63106
NTNYEEIEKV	1238.58992
TDYEEIEKVL	1238.61507
NTDYEEIEKV	1239.57394
YENENGHIRL	1244.60182
LYENENGHIR	1244.60182
RDDNEDLRVL	1244.62295
NRDDNEDLRV	1245.58182
REQIEELSKN	1245.64336
NQDDEEDLRL	1246.5546
HRNPEEIPWA	1248.612
NYEEIEKVLL	1249.66744
DYEEIEKVLL	1250.65146

KGRYEEIVKE	1250.67393
RSDQENPFIF	1252.59568
LLEEHEKETQ	1255.61647
SRSDYVYLPR	1255.64296
MTDEEFAREM	1258.50785
YEEIEKVLLE	1264.66711
DKEEEQEEET	1265.50156
REEDEEQVDE	1277.51279
LEEHEKETQH	1279.59132
REPDNRYDSE	1280.55018
FNTDYEEIEK	1287.57394
LEEHEKETHH	1288.59165
NQLDQMPRRF	1304.65281
RKEDDEEEEQ	1306.53934
REEDDEEEEQ	1307.48697
EHEEEEEQEQ	1315.49206
REKEDEEEKQ	1319.60736
DEEEEEEREQ	1321.50262
EDEERQPRHQ	1323.60362
DKNDEWHRVE	1327.60255
KIREEYPDRM	1336.6678
NRWFKIGFKR	1351.77458
ERKEEFFFPY	1391.66303
RWEREEDEEQ	1405.59786
LVIPVNGPGKF	1140.67756
DLGNPDSGENH	1154.47087
ERGDTIKIPAG	1156.63206
AIPVNKPGQLQ	1164.67353
DYAPGTSNDKV	1166.53241
TLAAPLNEIVE	1169.64123
SHNPGGPNEDF	1170.48104
KIGNPLEEGTL	1170.63648
IVDPGDSDIIK	1171.62049
GDSIGEEVEKL	1175.57902
LGEDPRGIPNN	1181.59093
LERGDAIKLPA	1182.6841
DEGSEPRVPAQ	1184.55421
DKIKEKLPGTG	1185.68376
LKSSKGELETV	1190.66269
AIPVNRPGQLQ	1192.67968
TIPVNKPGQLQ	1194.6841
GRPFIPDIPGK	1196.67862
DLGNPDHGEHL	1203.53889

VNEGKGDFELV	1206.60009
VEDGLHIISPE	1208.61574
KINPDAPLDKV	1209.68376
LERGDTIKLPA	1212.69466
SNKDKIILGPK	1212.73105
KFPPSVPSDKL	1214.67795
YDEGSEPRVPA	1219.55896
ATDDQIMDGVR	1220.55758
DGLHIISPELQ	1221.64738
PIEKEVEEGKA	1228.64196
NEDEEKGAIVK	1231.61647
KIHPDAPLDKV	1232.69975
KNIENYGLAVL	1233.68376
DMQDDGADEIK	1236.50487
ATDDQIMDGVR	1236.55249
YGPTPVRDGFK	1236.63715
GPKKIADMFPF	1250.66019
NLERGDTIKLP	1255.70048
DKIKEKIPGTE	1257.70489
KGELETVLDEQ	1260.63179
GHYPNPDIEYG	1261.54839
GEEEERSSESQ	1266.50804
KPRTDLPNVVE	1267.70048
RRLLPEEGTAE	1270.67499
EGEQIDRALLK	1271.69539
MDKIKEKLPGT	1275.6977
GINAENNQRNF	1276.60289
GINAENNERNF	1277.5869
TDDQIMDGVRK	1277.61543
LHPIKDVPSEF	1281.68376
GSETYPYPRRG	1282.61747
RSPQDERGQIV	1284.66549
NSLTLPVLRYL	1288.76235
SSNNQLDQMPR	1289.59027
SLSDRFSYVAF	1291.63173
TVLKPDDRNSF	1291.66409
TLPILRNLRLS	1295.81578
GDYWEDIGTIK	1296.61066
EITPEKNPQLQ	1296.67941
AFLSPHHYDSE	1302.57494
KGQTPLFPRIF	1303.75212
KDDEMSFLPIL	1307.65517
SPNDRNSYNLE	1308.58148

PKEEIKKPDVK	1310.76783
ANRDDNEDLRV	1316.61893
GKEIEKEPKFL	1317.74128
EITPEKNQQLQ	1327.68522
KNPQLQDLDIF	1330.70014
EEQGEEEINKQ	1332.59138
NTEEDTAKRLR	1332.68662
VLDEQKKGRIF	1332.76341
NLRSRNPIYSN	1333.69712
REGEEEERSSE	1336.56114
ETWNPNNPELK	1341.64336
FENENGHIRLL	1341.69097
RSPRDERSQIV	1342.71859
REEELNNLRGD	1344.65023
GFKRDFLEDAF	1344.65828
VNQDDEEDLRL	1345.62301
EITPEKKYPQL	1345.73619
YVPRDENFGHL	1346.64878
ELEKEEEEEEG	1349.55908
AEKVDEVFERK	1349.70596
KGRYEEIVKEV	1349.74234
TNYEEIEKVLL	1350.71512
AREPDNRYDSE	1351.5873
NTNYEEIEKVL	1351.67399
DKEEEQEEETS	1352.53359
NRDDNEDLRVL	1358.66588
DEKLRQDSELQ	1360.6703
HIDWKETPEAH	1362.64369
DLAKNKNQYLR	1362.74882
EEQVDEEWRGS	1363.57606
ETWNPNHPELK	1364.65934
RVDWKETPEAH	1367.67024
GDLKDKPWWPK	1369.7263
EVDRILENQKQ	1371.72267
RSDQENPFIFK	1380.69064
RSRNPIYSNKF	1381.73351
FNTDYEEIEKV	1386.64235
RQLPRQPSELY	1386.74882
HYPNPDIEYGW	1390.60624
DNEERKEEHGR	1398.63564
DRERIPERVVH	1405.76587
EREEDEEQVDE	1406.55539
DEDEEKQPRHQ	1410.62441

NNQLDQMPRRF	1418.69574
LREEDDEEEEQ	1420.57104
DEEEEQREEET	1422.5503
REDPEERARLR	1426.75095
RREDPEERARL	1426.75095
EDDEEEEQREE	1436.52957
VQPGRERWERE	1441.72948
EVQPGRERWER	1441.72948
EHEEEEEQEQE	1444.53465
EDEEEEEEREQ	1450.54522
RKEEEEEEQEQ	1462.62922
EEEEEREQRHR	1526.69422
REGDIIAVPTGI	1240.68958
DKIKEKLPGTGA	1256.72088
SIVDPGDSDIIK	1258.65252
HVPVLAPLPIGF	1259.75106
SNGNRGPLVQPQ	1266.65492
NDLGNPDSGENH	1268.5138
LERGDTIKLPAG	1269.71613
GPTPVRDGFKSL	1273.68991
AKINPDAPLDKV	1280.72088
SKKGVSSESEPF	1281.63212
DLAIPVNRPGQL	1292.73211
NLERGDAIKLPA	1296.72703
GKSEPEGEVPEF	1304.60049
GGNPETEFPETQ	1305.55935
LAIPVNRPGQLQ	1305.76375
AFPGSSHEVDRL	1314.64369
GRNEDEEKGAIV	1316.64408
NDLGNPDHGEHL	1317.58182
GGNPEIEFPETQ	1317.59574
FESAGRKPESVL	1319.69539
ILYPGDESIEGK	1320.66817
RTVPAPKRGEIV	1322.79029
YGPTPVRDGFKS	1323.66918
NLERGDTIKIPA	1326.73759
DAPELIKPPKIL	1333.80896
LDKIKEKLPGGH	1334.77906
KVEDGLHIISPE	1336.71071
VEQPIVEEPEAV	1338.67874
YDEGSEPRVPAQ	1347.61753
ATDDQIMDGVRK	1348.65254
SKKSLPSEFEPI	1361.73111

KSNNPFKFLVPA	1361.7576
LGSEEEEEAKDQ	1363.58596
DEIDAAPEERAR	1371.6499
RKDPNSEKPATE	1371.68628
IVEEDDKPSKIV	1371.73659
EEPQESEQGEGR	1374.57679
VMDKIKEKIPGT	1374.76611
SLNTKEDTAKRL	1375.75397
VTVPKEEVKKPE	1382.78896
DKIKEKIPGTEQ	1385.76347
DALEPDNRIESE	1387.63358
KDTDSEEELKEA	1393.63291
LRSPQDERGQIV	1397.74955
SLPSEFEPINLR	1401.73726
SGKEIEKEPKFL	1404.77331
GQGEEEEELEKE	1405.59652
RSPQDERGQIVK	1412.76045
REGEEEERSSES	1423.59317
GLDDERAKEMPY	1423.65221
ANRDDNEDLRVL	1429.703
VNQDDEEDLRVV	1430.67578
SGFKRDFLEDAF	1431.69031
TETWNPNNPELK	1442.69103
VNQDDEEDLRLV	1444.69143
GHYPNPDIEYGW	1447.62771
MDEVDKYIPIPQ	1447.71374
REKKEQKEVQPG	1455.79142
LRSPRDERSQIV	1455.80265
LVNQDDEEDLRL	1458.70708
AEPEQKEEESQR	1459.66594
KDSLCFIEVIVH	1459.76136
TETWNPNHPELK	1465.70702
NTDYEEIEKVLL	1465.74207
TRVDWKETPEAH	1468.71792
RQEINEENVIVK	1470.79108
SKDLINWKELQP	1470.7951
EITPEKKYPQLQ	1473.79477
GLREEDDEEEEQ	1477.5925
RGSRQEEEEDED	1478.59898
GDLKDKPWWPKL	1482.81036
AEREIVRDIKEK	1485.83837
SEKEEEDEDEPR	1491.60815
FNTNYEEIEKVL	1498.7424

FNTDYEEIEKVL	1499.72642
KKYPQLQDLDLF	1507.81551
DLFENLQNYRLV	1523.78527
EKNWPPFFPIIH	1524.7998
KEEEDEDEPRSY	1525.62889
EREEDEEQVDEE	1535.59798
DEEEEQREEETK	1550.64527
VQPGRERWEREE	1570.77208
EVQPGRERWERE	1570.77208
RREDPEERARLR	1582.85206
REKEDEEEKQKY	1610.76566
VTPGSDAPKVAPE	1267.65286
ADPDDLGKGGHEL	1323.61753
KGDVIAIPPGIPY	1339.76201
RVTPQPGVPPEEA	1376.71685
NLERGDTIKIPAG	1383.75905
GDAKPIEKEVEEG	1400.69036
AFPGSAQEVDRIL	1402.7325
DLAIPVNRPGQLQ	1420.79069
DLTIPVNKPGQLQ	1422.7951
VIGEPIDEKGELK	1426.77879
LGGNPEIEFPETQ	1430.6798
YGPTPVRDGFKSL	1436.75324
RESPEATKPADEL	1442.71216
GRNEDEEKGAIVK	1444.73905
GIMDKIKEKLPGT	1445.80323
VEDGLHIISPELQ	1449.75838
NYDEGSEPRVPAQ	1461.66046
GEEDNVISQVERP	1471.70233
EAEDVFSKITPKD	1478.73732
EVKKPEAKPIQIT	1480.87336
RADLYNPRAGRIS	1488.80298
YNLERGDTIKIPA	1489.80092
VDAPDMERSPINF	1490.6944
TVPKEEIKKPDVK	1510.88392
RSPQDERGQIVKV	1511.82886
RIPPTEEIADRIN	1523.81763
DLPVLRWLKLSAE	1539.88934
REGEEEERSSESQ	1551.65175
RLRSPQDERGQIV	1553.85066
EFVNPKEHDKPVQ	1566.79108
AEPEQKEEESQRK	1587.7609
EEEEQGEEEINKQ	1590.67657

SSNNQLDQMPRRF	1592.7598
KEDDEEEEQGEEE	1594.58748
EDEEQVDEEWRGS	1607.6456
RLRSPRDERSQIV	1611.90376
FNTDYEEIEKVLL	1612.81048
EKEEEDEDEPRSY	1654.67148
DEEEEQREEETKN	1664.68819
DDEEEEQREEETK	1665.67221
EEDEDEDEERQPR	1675.66779
EEEEDEDEERQPR	1689.68344
KEVQPGRERWERE	1698.86704
REEDDEEEEQREE	1721.67327
YEKEEDEEEKQKY	1746.77047
RWEREEDEEQVDE	1748.73581
SKGDVIAIPPGIPY	1426.79404
KAKLSPGDVFVIPA	1441.84133
PGAFADLKSEQLGQ	1460.73798
DVTHPHPGEDSSPS	1461.62408
RAKLSPGDVFVIPA	1469.84747
GIMDKIKEKLPGTG	1502.82469
GDAKPIEKEVEEGK	1528.78533
KGDAKPIEKEVEEG	1528.78533
SNNPAFSNKFGSLF	1529.73832
GVQRGLVGEIISRF	1530.87509
GRNEDEEKGAIVKV	1543.80746
LEPDHRVESEAGLT	1552.76018
DRETGNVANFVTSF	1556.73396
GVMDKIKEKIPGTE	1560.83017
SYNLERGDTIKIPA	1576.83295
KVEDGLHIISPELQ	1577.85335
YLGGNPETEFPETQ	1581.70674
YLGGNPEIEFPETQ	1593.74313
DNVISQIENPVKEL	1597.84318
KLPPPEPKKPKTIQ	1600.97849
EEVKKPEAKPIQIT	1609.91595
VTVPKEEIKKPDVK	1609.95233
SHGQGEEEEELEKE	1629.68746
EEPQESEQGEGRRY	1693.74123
KKYPQLQDLDLFVS	1693.91595
EFVNPKEHDKPVQQ	1694.84966
KDKRQQSQEENVIV	1700.89259
DKEEEQEEETSKQV	1707.75554
GEKEEKRHGEWRPS	1724.84631

SEKEEEDEDEPRSY	1741.70351
EDEEEGQRERGRQE	1746.76376
RKEDDEEEEQGEEE	1750.68859
KKEDEDEDEEEEEE	1781.67193
DEEEEQREEETKNQ	1792.74677
YEKEEDEEEGQRER	1825.78349
KEVQPGRERWEREE	1827.90963
REEDDEEEEQREEE	1850.71586
RWEREEDEEQVDEE	1877.7784
APVSEGGGEPPKESL	1453.71691
DAPRPGQPGQAPRPA	1514.78225
NIVSALDKAAKEVAF	1575.87408
TKGDAKPIEKEVEEG	1629.83301
SPRIPPTEEIADRIN	1707.90242
TVTVPKEEIKKPDVK	1711.00001
DDNVISQIENPVKEL	1712.87012
ITEPEVPEKEEPKAE	1724.85889
AFLSPHHYDSEAILF	1746.8486
KVSREQIEELSKNAK	1758.97084
IINPKGKEEEEEKEQ	1799.90215
KEDDEEEEQGEEEIN	1821.71447
DKEEEQEEETSKQVQ	1835.81412
SKKEDEDEDEEEEEE	1868.70396
KDRRQEINEENVIVK	1870.0141
DDEEEEQREEETKNQ	1907.77371
EEDEDEDEERQPRHQ	1940.78528
KEVQPGRERWEREED	1942.93658
REEDDEEEEQREEET	1951.76354
RQEEDEDEEKQPRHQ	1952.86929
EEEEDEDEERQPRHQ	1954.80093
RQEEDEDEDEERQPR	1959.82748
RQEEEEDEDEERQPR	1973.84313
REEEEEEEEDEEKQR	1992.82648
HMPPSSGSAPVNLEPF	1666.78937
GDAKPIEKEVEEGKAK	1727.9174
SDDNVISQIENPVKEL	1799.90215
TITEPEVPEKEEPKAE	1825.90657
VGRRGGQQQEEESEEQ	1845.83217
RIINPKGKEEEEEKEQ	1956.00326
RKEDDEEEEQGEEEIN	1977.81558
KHSEKEEEDEDEPRSY	2006.85738
EQRKEDDEEEEQGEEE	2007.78976
SRQEEDEDEEKQPRHQ	2039.90131

SRQEEDEDEDEERQPR	2046.85951
SRQEEEEDEDEERQPR	2060.87516
KEDEDEDEEEEEEREQ	2066.77925
REEEEEEEEDEEKQRS	2079.85851
REEDDEEEEQREEETK	2079.85851
KEQKEVQPGRERWERE	2084.06317
HREEEEEEEEDEEKQR	2129.88539
RDDAPRPGQPGQAPRPA	1785.9103
DALEPDNRIESEGGLIE	1856.88723
GSDDNVISQIENPVKEL	1856.92361
RRDDAPRPGQPGQAPRP	1870.9743
VTVPKEEVKKPEAKPIQ	1920.11644
GEEDNVISQVQRPVKEL	1940.00834
LVGRRGGQQQEEESEEQ	1958.91624
RKDPNSEKPATETYVPR	1988.01958
KRESHGQGEEEEELEKE	2042.92613
GEEEERSSESQERRNPF	2065.91696
RGSRQEEDEDEDEERQP	2103.88097
GRQEGEKEEKRHGEWRP	2108.03802
GSRQEEEEDEDEERQPR	2117.89662
SEKEEEDEDEPRSYETR	2127.89489
EKEEDEEEGQRERGRQE	2132.94391
KEEEEEEEEDEEEKQRS	2180.89495
LREEDDEEEEQREEETK	2192.94257
REEDDEEEEQREEETKN	2193.90143
KKEDEDEDEEEEEEREQ	2194.87421
REEEEEEEEDEEKQRSE	2208.9011
HREEEEEEEEDEEKQRS	2216.91742
RQEEDEDEDEERQPRHQ	2224.94497
RQEEEEDEDEERQPRHQ	2238.96062
AITGAAISTAANLLTGIF	1704.95306
AITGAAIATAAEFINYLT	1810.95854
GTGLIKAIEGYVLANPGY	1835.99018
RRDDAPRPGQPGQAPRPA	1942.01141
TKGDAKPIEKEVEEGKAK	1957.06005
DLPVLRWLKLSAEHGSLH	2071.14472
RRGGQQQEEESEEQNEGN	2103.89221
KKRESHGQGEEEEELEKE	2171.02109
GRQEGEKEEKRHGEWRPS	2195.07005
RNNDEKLRNRHGPVEMPY	2225.09924
RKEDDEEEEQGEEEINKQ	2233.96912
RNNDEKLRNRHGPVEMPY	2241.09416
GLREEDDEEEEQREEETK	2249.96403

RGSRQEEDEDEDEERQPR	2259.98208
RGSRQEEEEDEDEERQPR	2273.99773
SKKEDEDEDEEEEEEREQ	2281.90624
YEKEEDEEEGQRERGRQE	2296.00723
SHREEEEEEEEDEEKQRS	2303.94945
SRQEEDEDEDEERQPRHQ	2311.977
REEDDEEEEQREEETKNQ	2321.96001
SRQEEEEDEDEERQPRHQ	2325.99265
HREEEEEEEEDEEKQRSE	2345.96001
SDEESDEDSSSDEEAPKAK	2054.81564
IRRRDDAPRPGQPGQAPRP	2140.15947
NLPLDVVAATFNLQRNEAR	2141.14618
GRRGGQQQEEESEEQNEGN	2160.91367
EKKRESHGQGEEEEELEKE	2300.06369
EKERESHGQGEEEEELEKE	2301.01132
EKEEDEEEGQRERGRQEGE	2319.00796
REGEEEERSSESQERRNPF	2351.06067
GSRQEEEEDEDEERQPRHQ	2383.01411
SHREEEEEEEEDEEKQRSE	2432.99204
SKKEDEDEDEEEEEEREQR	2438.00735
REEEEEEEEDEEKQRSEER	2494.0448
KEDEDEDEEEEEEREQRHR	2516.04039
SSDEESDEDSSSDEEAPKAK	2141.84767
IRRRDDAPRPGQPGQAPRPA	2211.19659
VGRRGGQQQEEESEEQNEGN	2259.98208
NLPLDVVAATFNLQRNEARQ	2269.20475
AEKKRESHGQGEEEEELEKE	2371.1008
EDEEEGQRERGRQEGEKEEK	2447.10293
ERGRQEGEKEEKRHGEWRPS	2480.21375
YEKEEDEEEGQRERGRQEGE	2482.07129
RGSRQEEDEDEDEERQPRHQ	2525.09957
RGSRQEEEEDEDEERQPRHQ	2539.11522
HREEEEEEEEDEEKQRSEER	2631.10371
NIENYGLAVLEIKANAFLSPH	2313.22376
LVGRRGGQQQEEESEEQNEGN	2373.06615
NLPLDVVAATFNLQRNEARQL	2382.28882
TAEKKRESHGQGEEEEELEKE	2472.14848
EEQEKDRKRRQQGEETDAIVK	2572.30738
EKEEDEEEGQRERGRQEGEKE	2576.14552
RERGRQEGEKEEKRHGEWRPS	2636.31487
QRGSRQEEEEDEDEERQPRHQ	2667.1738
SHREEEEEEEEDEEKQRSEER	2718.13574
SKKEDEDEDEEEEEEREQRHR	2731.16738

REEEEEEEEEDEEKQRSEERKN	2736.18269
RATPAEVLANAFGLRQRQVTEL	2440.34192
VINNLPLDVVAATFNLQRNEAR	2467.34158
LEEQEKDRKRRQQGEETDAIVK	2685.39144
YEKEEDEEEGQRERGRQEGEKE	2739.20885
EDEEEGQRERGRQEGEKEEKRH	2740.26295
SVINNLPLDVVAATFNLQRNEAR	2554.37361
VINNLPLDVVAATFNLQRNEARQ	2595.40016
NLPLDVVAATFNLQRNEARQLKS	2597.41581
SGFSKNILEAAFNTNYEEIEKVL	2616.31917
EDEEEGQRERGRQEGEKEEKRHG	2797.28441
SHREEEEEEEEDEEKQRSEERKN	2960.27363
SVINNLPLDVVAATFNLQRNEARQ	2682.43219
VINNLPLDVVAATFNLQRNEARQL	2708.48422
NLPLDVVAATFNLQRNEARQLKSN	2711.45874
SKNILEAAFNTNYEEIEKVLLEQQ	2823.44108
SVINNLPLDVVAATFNLQRNEARQL	2795.51625
VAKPKPGSSVAIFGLGAVGLAAAEGA	2338.31291
TVDNLTAFLGRSVSLQLISATKPDAT	2718.46723
SVINNLPLDVVAATFNLQRNEARQLK	2923.61121
KNPQLQDLDIFVNSVEIKEGSLLLPH	2946.5935
SVINNLPLDVVAATFNLQRNEARQLKS	3010.64324
NLPLDVVAATFNLQRNEARQLKSNNPF	3069.62284
SGFSKNILEAAFNTNYEEIEKVLLEQQ	3114.56299
GSTVDNLTAFLGRSVSLQLISATKPDAT	2862.52073
TVDNLTAFLGRSVSLQLISATKPDATGK	2903.58366
GSTVDNLTAFLGRSVSLQLISATKPDATGK	3047.63715
VINNLPLDVVAATFNLQRNEARQLKSNNPF	3395.81825
TVPQNYAVAAKSLSDRFSYVAFKTNDRAGIA	3360.73351
SVINNLPLDVVAATFNLQRNEARQLKSNNPF	3482.85027
EITPEKNPQLQDLDIFVNSVEIKEGSLLLPH	3515.86319
